# Areas of improvement in anticoagulant safety. Data from the CACAO study, a cohort in general practice

**DOI:** 10.1371/journal.pone.0175167

**Published:** 2017-04-06

**Authors:** Paul Frappé, Joël Cogneau, Yoann Gaboreau, Nathan Abenhaïm, Marc Bayen, Matthieu Calafiore, Claude Guichard, Jean-Pierre Jacquet, François Lacoin, Laurent Bertoletti, CACAO study investigators

**Affiliations:** 1 Department of General Practice, University of Saint-Etienne, Saint-Etienne, France; 2 Inserm U 1059, Sainbiose DVH, University of Saint-Etienne, Saint-Etienne, France; 3 CIC-EC 1408, Saint-Etienne, France; 4 Institut de Recherche en Médecine Générale, Paris, France; 5 Department of General Practice, Grenoble Alpes University, Grenoble, France; 6 TIMC-IMAG UMR 5525, Grenoble Alpes University, Grenoble, France; 7 Department of General Practice, University of Lille, Lille, France; 8 Department of Vascular Medicine and Therapeutics, CHU de Saint-Etienne, Saint-Etienne, France; Maastricht University Medical Center, NETHERLANDS

## Abstract

**Background:**

Real-world studies on anticoagulants are mostly performed on health insurance databases, limited to reported events, and sometimes far from every-day issues in family practice. We assess the presence of data for safe monitoring of oral anticoagulants in general practice, and compare patients’ knowledge of taking an anticoagulant between vitamin K antagonists (VKA) and direct anticoagulants (DOAC), and the general practitioner’s perception of their adherence to anticoagulation.

**Methods:**

The CACAO study is a national cohort study, conducted by general practitioners on ambulatory patients under oral anticoagulant. In the first phase, investigators provided safety data available from medical records at inclusion. They also evaluated patients’ knowledge about anticoagulation and graded their perception of patients’ adherence.

**Results:**

Between April and December 2014, 463 general practitioners included 7154 patients. Renal and hepatic function tests were respectively unavailable in 109 (7.5%) and 359 (24.7%) DOAC patients. Among patients with atrial fibrillation, 345 patients (6.9%) had a questionable indication of anticoagulant (CHA2DS2-Vasc<2). One hundred and thirty-three VKA patients (2.3%) and 70 DOAC patients (4.9%) answered they took no anticoagulant (p<0.0001). According to general practitioners’ perception, 430 patients (6.1%) were classified as “not very” or “not adherent”, with no difference between groups.

**Conclusions:**

Our results highlight the efforts needed to improve anticoagulant safety in daily practice: decreasing the rate of unknown biological data in patients with DOACs or the rate of patients with VKA with no strong indication of anticoagulation, and improving patient knowledge with regard to their anticoagulant. Patients’ adherence seems highly over-estimated by the general practitioners.

**Clinical trial registration:**

ClinicalTrials.gov NCT02376777

## Introduction

In ambulatory care, anticoagulants are involved in 12% of suspected adverse drug reactions (ADR) [1]. In older patients, they constitute the first drug implicated in suspected ADR [1] and the first cause of admission to emergency department for an ADR [2]. Inadequate drug monitoring and ignoring clinical or laboratory results are the most frequent omission errors leading to a preventable ADR in an ambulatory context [3].

Oral anticoagulants are widely prescribed in family practice. Their main indications are atrial fibrillation and venous thromboembolic disease. In France, 2.1% of the population had an oral anticoagulant in 2013 [4]. Since their introduction in 2009, the proportion of direct oral anticoagulants (DOAC) is increasing constantly [5].

Several real-world studies provided results confirming those of phase III studies on DOAC, showing at least similar efficacy and safety [6–9]. However, these studies are mostly performed on health insurance databases, limited to reported events. Other practical issues on anticoagulant management by general practitioners remain. Having available renal and hepatic function tests (in order to adjust drug and regimen choices), performing coagulation tests when appropriate, informing patient, inquiring about adherence and potential interactions are all tasks assigned to the general practitioner, as well as all potential sources of ADR [10,11].

The primary aim of this study was to assess in general practice records the presence of mandatory data for safe monitoring of oral anticoagulants. The secondary objectives were to compare between vitamin K antagonists (VKA) and DOAC, patient knowledge of taking an anticoagulant, and the perception by the general practitioners of their adherence to anticoagulation.

## Materials and methods

The CACAO study (Comparison of Accidents and Circumstances with Oral Anticoagulants) is a national cohort study, conducted by general practitioners from all over France on ambulatory patients with an oral anticoagulant. These 463 investigators covered 290 different rural or urban towns, and were distributed over 47 different departments of France. This study was approved by the ethical committee of the University Hospital of Saint-Etienne (IRBN112014/CHUSTE). Its protocol has been registered in ClinicalTrials.gov (NCT02376777). All patients received written information about the study, emphasizing their right to refuse participation, or to withdraw at any time. No written informed consent was required for inclusion. This cohort is divided into two distinct periods: phase 1 (baseline data) and phase 2 (follow-up data). We report the phase 1 results here.

### Study population

Every patient aged 18 years or more, taking an oral anticoagulant, and consulting a general practitioner investigator, whatever his or her reason for consultation, were included. Patients with injectable anticoagulants and those under the age of 18 were not included. Each investigator included all consecutive eligible patients for 3 months, beginning inclusions between April and October 2014.

### Data collection

When the patient was eligible, the general practitioner first asked a standardized question, using the following formulation: “Can you tell me if, among your medications, there is an anticoagulant?”. Then the general practitioner collected demographic status, personal history, current medications, items of CHA2DS2-Vasc and HAS-BLED scores [12,13] and results of available biological tests performed (renal and hepatic functions, coagulation tests). Co-medication list was built from the ANSM (National agency for medication safety) list [4]. No specific biological sample was asked for this study, as our aim was to assess the prevalence of information available from the patient’s medical record. The general practitioner was finally asked to rate his perception of the patient’s adherence to anticoagulant. Data were collected anonymously by physicians via an e-CRF.

### Statistical analysis

We used descriptive statistics with a 95% confidence interval (CI) to analyze the prevalence of available information, patients’ knowledge and their adherence to treatment as perceived by the general practitioners. Normality tests have been performed with quantitative variables. In case of normality, these variables were described by their mean and standard deviation. In other cases, they were described by their median and interquartile range, Q1-Q3. Data were secondarily compared between VKA and DOAC groups, using Chi-squared or Fisher’s exact test for qualitative data, and Student’s t-tests or rank tests for quantitative data. We performed analysis using IBM SPSS Statistics 20® (International Business Machines Corp).

## Results

Between April and December 2014, the 463 general practitioners included 7154 patients: 5699 (79.7%) with a VKA and 1455 (20.3%) with a DOAC. For 6824 patients (95.4%), the investigator was their usual physician. Table 1 shows the patients’ characteristics.

**Table 1:** Characteristics of patients.

Characteristics		VKA	DOAC	p
Patients, No. (%)		5699 (79.7)	1455 (20.3)	
Age				
	median (IQR), y	78 (69–84)	75 (67–82)	<0.0001
	≥75 y, No. (%)	3516 (61.7)	763 (52.4)	<0.0001
Male, No. (%)		3083 (54.1)	764 (52.5)	0.278
Weight, median (IQR), kg		76 (66–88)	78 (66–90)	0.099
BMI, median (IQR), kg/m^2^		27.3 (24.3–31.1)	27.7 (24.5–31.2)	0.103
Personnal history, No. (%)				
	Hypertension	3872 (67.9)	960 (66.0)	0.154
	DVT and/or PE	1514 (26.6)	239 (16.4)	<0.0001
	Diabetes mellitus	1307 (22.9)	315 (21.6)	0.296
	Coronaropathy and/or MI	1178 (20.7)	210 (14.4)	<0.0001
	Symptomatique heart failure	1154 (20.2)	188 (12.9)	<0.0001
	Stroke and/or TIA	931 (16.3)	219 (15.1)	0.234
	Peripheral arterial disease	490 (8.6)	72 (4.9)	<0.0001
	Hemorrage requiring hospitalization	477 (8.4)	68 (4.7)	<0.0001
Anticoagulant, No. (%)				
	Fluindione	4161 (73.0)	-	
	Warfarine	1112 (19.5)	-	
	Rivaroxaban	-	823 (56.6)	
	Dabigatran	-	544 (37.4)	
	Acenocoumarol	426 (7.5)	-	
	Apixaban	-	88 (6.0)	
Indication for anticoagulation, No. (%)				
	Valvular atrial fibrillation	594 (10.4)	60 (4.1)	<0.0001
	Non-valvular atrial fibrillation	3274 (57.4)	1111 (76.4)	<0.0001
	DVT/PE	1257 (22.1)	208 (14.3)	<0.0001
	Surgery	36 (0.6)	29 (2.0)	<0.0001
	Heart valve prothesis	524 (9.2)	5 (0.3)	<0.0001
	Other	459 (8.1)	83 (5.7)	0.003
	Unknown	13 (0.2)	1 (0.1)	0.326
Duration of anticoagulant treatment >1 year, No. (%)		4848 (85.1)	788 (54.2)	<0.0001
CHA2DS2- Vasc / atrial fibrillation (n = 5039)				
	0	44 (1.1)	40 (3.4)	<0.0001
	1	187 (4.9)	74 (6.3)	
	≥2	3637 (94.0)	1057 (90.3)	
HASBLED / atrial fibrillation (n = 5039)				
	≤3	3017 (78.0)	1040 (88.8)	<0.0001
	>3	851 (22.0)	131 (11.2)	

VKA: vitamine-k antagonists, DOA: direct oral anticoagulants, BMI: body mass index, DVT: deep vein thrombosis, PE: pulmonary embolism, MI: myocardial infarction, TIA: transient ischemic attack

Prevalence of information available in patients’ records are shown in Table 2. In patients with DOAC, renal function tests were not available in 109 (7.5% (95% CI, 6.1%-8.9%)) cases, and for hepatic function in 359 cases (24.7% (95% CI, 22.5%-26.9%)). In patients anticoagulated for atrial fibrillation, the CHA2DS2-Vasc score was 0 or 1 in 345 cases (6.9% (95% CI, 6.1%-7.5%)).

**Table 2:** Safety data in patients’ records.

Data, No. (%)		VKA (n = 5699)	DOAC (n = 1455)	p
Date of the last INR				
	<1 month	5039 (91.4)	-	
	1–3 months	391 (7.1)	-	
	>3 months	84 (1.5)	-	
Renal failure				
	No failure (clearance ≥ 60 mL/min)	3506 (61.5)	1037 (71.3)	
	Moderate (30 mL/min ≤ clearance < 60 mL/min)	1549 (27.2)	301 (20.7)	
	Severe (15 mL/min ≤ clearance < 30 mL/min)	175 (3.1)	8 (0.5)	
	Terminal (clearance < 15 mL/min)	13 (0.2)	0 (0.0)	
	*Unknown*	*456 (8*.*0)*	*109 (7*.*5)*	*0*.*549*
Hepatic function				
	AST and/or ALT >3N	29 (0.5)	3 (0.2)	
	Bilirubin >2N	4 (0.1)	1 (0.1)	
	*Unknown*	*1645 (28*.*9)*	*359 (24*.*7)*	*0*.*002*
Concomitant medications				
	Statin	2310 (40.5)	561 (38.6)	
	Amiodaron	829 (14.5)	263 (18.1)	
	Antiplatelet	599 (10.5)	115 (7.9)	
	Serotonin reuptake inhibitors	328 (5.8)	85 (5.8)	
	Fibrate	154 (2.7)	35 (2.4)	
	Vérapamil	134 (2.4)	33 (2.3)	
	NSAID	56 (1.0)	29 (2.0)	
	Quinidine	14 (0.2)	4 (0.3)	
	Carbamazepine	12 (0.2)	2 (0.1)	
	Tacrolimus	11 (0.2)	1 (0.1)	
	Ciclosporin	7 (0.1)	0 (0.0)	
	Anticoagulant	6 (0.1)	0 (0.0)	
	Systemic antifungal therapy	2 (0.0)	0 (0.0)	
	Rifampin	1 (0.0)	0 (0.0)	
	Protease inhibitors	1 (0.0)	0 (0.0)	
	*At least 1 medication*	*3310 (58*.*1)*	*836 (57*.*5)*	*0*.*677*

INR: international normalized ratio, AST: aspartate transaminase, ALT: alanine transaminase, NSAID: non-steroidal anti-inflammatory drug

At least one medication potentially interactive with anticoagulants was reported for 4146 patients (58.0% (95% CI, 56.9%-59.1%)). Besides the suggested treatments, general practitioners reported 100 other treatments as being at risk of interaction, such as proton-pump inhibitors, diuretics and hemigoxin.

Patients under DOAC responded more frequently “No” to the question “Do you take an anticoagulant?” than patients under VKA (Table 3).

**Table 3:** Patient’s knowledge of taking an anticoagulant.

Variable, No. (%)		VKA (n = 5642)	DOAC (n = 1437)	p
"Do you take an anticoagulant?"				
	Yes	5083 (90.1)	1248 (86.8)	<0.0001
	No	131 (2.3)	70 (4.9)	
	Do not know	428 (7.6)	119 (8.3)	

General practitioners perceived the patient as hardly or not adherent in 430 cases (6.1% (95% CI, 5.5%-6.7%)) (Table 4).

**Table 4:** General practitoner’s perception of patient’s adherence to anticoagulation.

Variable, No. (%)		VKA (n = 5644)	DOAC (n = 1429)	p
Patient adherence as perceived by GP				
	Not adherent	41 (0.7)	13 (0.9)	0.224
	Not very adherent	288 (5.1)	88 (6.2)	
	Rather adherent	1856 (32.9)	486 (34.0)	
	Completely adherent	3459 (61.3)	842 (58.9)	

GP: general practitioner

## Discussion

We report the results of the first French national cohort study of ambulatory patients under oral anticoagulant therapy, a study designed and conducted by and for general practitioners. Our results emphasize the efforts needed to improve anticoagulant safety in daily practice: decreasing the rate of unknown biological data in patients with DOACs (renal and hepatic functions) or the rate of patients with VKA but with no formal indication of anticoagulation, and improving patient knowledge with regard to their anticoagulant therapy.

Our data show large differences with patients’ characteristics in phase III trials. Patients are older (mean age = 73 years in DOAC group vs 55 to 57 years in trials) with a higher rate of renal impairment (21.2% of patients had a clearance<60mL/min vs 1 to 8% with a clearance <50mL/min in trials), highlighting the need to evaluate safety issues in “real-world” studies [14–17]. Efforts are needed to increase the presence of biological data in DOACs patients: in patients with VKA, 1.5% had a date of last INR of over 3 months, whereas in DOACs patients, general practitioners did not know the renal function tests in one of 13 patients or the hepatic function tests in one of 4 patients. These data are particularly important: severe renal impairment is a contra-indication for all DOACs, moderate renal impairment implies adapting DOAC dosages, and hepatic impairment is a contra-indication for rivaroxaban and apixaban. These findings probably reflect the difficulties of appropriation of the monitoring modalities of this new pharmacological class.

Several situations reveal contra-indications where the general practitioner has its role of stopping harmful treatment. This is for example the case for the 84 patients with atrial fibrillation (1.7%) having a CHA2DS2-Vasc score of 0, which is not an indication of anticoagulant.

At first sight, associations with a medication at risk of interaction are frequent and concern more than half patients with anticoagulants. Associated medications can be distinguished by their type of interaction. Pharmacokinetic interaction, which constitutes the highest risk of interaction, modifying the medication concentration, is mainly represented by statin and amiodarone, involved in about 55% of cases. These medications can however be justified in this population with cardiovascular history, and occur as often as in the populations included in the phase III studies [14–18]. The clinical impact of such interactions is debatable. Strong inhibitors/inductors such as antifungals, verapamil or quinidine, are rare (<5%). Pharmacodynamic interactions, with potential effect and no modification of compound concentration, are represented first by antiplatelet association. This occurred in 10% of cases, a frequency similar to that of the population of phase III trials [14–17]. This combination is associated with a 2-fold increase in the risk of major bleeding [19] and the recent 2016 ESC guidelines restrain indications of this combination [20]. The increased bleeding risk with selective serotonin reuptake inhibitor has recently been specified [21]. Prevalence of this combination (5.8%) matches the usual prescription data in France, where psychoanaleptics represent 2.1% of the drug sales market share [22]. Given these points, the data provided by our study do not ring alarm bells as to a large risk of interactions in daily practice. Efforts should probably be more focused on the one hundred supplementary treatments stated by the physicians that highlight the difficulty of identifying the risk of interaction in a practical context. Simple alerts of “at-risk patients” should be developed, rather than software which usually indicates situations of at-risk “drug-drug interaction” irrespective of the risk. The follow-up of the CACAO cohort should provide valuable data on this topic.

With twice as many patients under DOAC as VKA who think they are not taking an anticoagulant, our data confirmed the presentiment of many physicians. From the patient’s point of view, the apparent simplicity of DOAC monitoring can give them the impression of a harmless treatment which, added to the drug novelty, make them forget they are taking anticoagulant therapy. Such oblivion potentiates the risk of this treatment, especially in an emergency context or when the physician does not know the patient and has no access to their usual treatment, leading for example to the prescription of contra-indicated drugs. Several studies have reported patient adherence to oral anticoagulation. Under VKA, the proportion of non-adherent patients varies between 22% and 58% [23]. Regarding DOACs, in phase III studies, adherence varied between 71% and 98% and the rate of discontinuation for non-adherence under DOAC was similar or even higher than warfarin [23]. Adherence of patients appears much higher in our study, which could be explained by its indirect measurement mode, based on the perception of the general practitioner. This less reliable method is however closer to real-world practice, where the physician does not have the tools to measure actual patient adherence, and can only go by his feelings to adapt the treatment. The one year results of the CACAO study should allow us to compare this adherence perceived by the physician to adherence auto-assessed by the patient and to the incidence of thromboembolic events.

The multicenter nature and the large size of the study may help improve the generalizability of the findings. Given that the investigator was the usual physician in 95% of cases, the data were easier to access, with easy filling in of the questionnaire. However, the data do remain declarative and not objectively verifiable, representing a potential measurement bias. It is possible that physicians who agreed to participate in the study were more sensitive to continuing medical education, medical information and/or to the anticoagulant topic than the average general practitioner. This possible recruitment bias should have a tendency to underestimate the result of our outcomes which are nevertheless significant for practice.

## Conclusions

In a context of appropriation of new monitoring practices, this study puts forward concrete ways of improvement, such as knowledge of the liver and kidney function in patients under DOAC, simplification of discourse on drug interactions, and patient information to minimize iatrogenic risks in real life. Moreover, patients’ adherence seems highly over-estimated by the general practitioners subjective perception, and may prompt general practitioners to use validate tools to assess patients’ adherence.

## Appendix

The members of the CACAO Study Group were as follows (n = 463, all in France):

Nathan Abenhaïm, Sophie Ackermann, Maryse Adam Blanpain, Xavier Andreu, Céline Arnould, Audrey Atlan-Cottin, Jean-Pierre Aubert, Isabelle Aubin-Auger, Jacques Aubry, Julien Augueux, Veena Augustin, Annick Bakry, Marine Baldesi, Eric Banoun, Eric Barberet, Rémi Bardet, Florence Barriere, Dan Baruch, Nicolas Baude, Marc Bayen, Sabine Bayen, François Bayle, Yannick Beaufils, Alain Beaupin, Julie Bedel-Chauvaud, Raphaël Bel, Martine Bellier, Farouk Bendamene, Philippe Berard, Cédric Berbé, Christophe Berkhout, Jacques Berland, Charles-Edouard Béthembos, Pierre-Yves Billiard, Olivier Bisch, Aurélie Bizeau, Paul Blanchard, Guy Blanquart, Aurélie Boch, Isabelle Bodein, Emmanuel Boige, Claude Bonin, Anne-Laure Bonis, Marie-Pierre Bonnard, Pascal Bonnet, Pierre-André Bonnet, Gérard Bosselut, Anne Bottet, Philippe Bouche, Bérengère Boucherle, Audrey Bougeard, Serge Bouhana, Mourad Boukeloul, Jean Boulet-Gercourt, Jean-Marie Boulongne, Lionel Bouniol, Jean-Jacques Bourcart, Michel Bourgoin, Véronique Bourguignon-Vartanian, Claire Bouteville, Philippe Boutin, Annelore Boutmy, Evelyne Brenner-Girault, Nicolas Breton, Muriel Briane-Fraysse, Marina Brodbeck, Olivier Brunet, Ariel Buchinger, Anne Buffaz-Sutra, Marc Bur, Philippe Cabourdin, Philippe Cachera, Eric Cailliez, Matthieu Calafiore, Denis Calvet, Pierre Camedescasse, Hervé Canart, Michel Cancade, Christophe Candelier, Christian Capiod, Thierry Carin, Olivier Caron, Elisa Carré, Yannick Carrillo, Nathalie Casagrande, Pierre Causse, Gaëlle Chabert, Frédérick Chabord, Juliette Chambe, Guillaume Chambon, Richard Champeaux, Laurent Charbonnel, Rodolphe Charles, Clément Charra, Samuel Chartier, Julie Chastang, Sophia Chatelard, Eric Chatillon, Laurent Chauvot, Hervé Chelle, Stéphane Chenuet, Nicolas Chevalier, David Chevillot, Jean-Pierre Cibeer, Jacques Cittée, Jean-François Claudel, Yvonnick Clemence, Jean-Paul Clerget, Isabelle Clusier-Jeudy, Marie-Pierre Coispeau, Arnaud Colin, Hervé Collart-Dutilleul, Laurence Compagnon, Marine Compan Malet, Laurent Connan, Hubert Conrad, Bruno Coquillaud, Jean-Luc Cormier, Jean-Charles Couette, Yves Cournoyer, Christian Cousin, Stephen Creton, Jean-Jacques Crignon, André Cros, Michel Cunin, Emmanuel Cussac, Jean-Maurice Dailly, Didier Danvin, Pierre-Marie Darnaut, Bruno Daubin, Thomas De L'Hamaide, Yves De Saint Meleuc, Sabine De Taddeo, Jean-Luc Decker, Anne Decobert, Yannick Delattre, Christine Delavenne, Loïc Delavenne, Bénédicte Delbru, Denis Deleplanque, Nicole Delerive, Laurent Delesalle, François Delforge, Jean-Paul Delgrange, Jean-Pierre Delpierre, Eric Demeulemeester, Nicolas Derain, Fabrice Descombe, Thierry Desmoulins, Marie-Claire Deville-Carollo, Jean-Michel Dherbecourt, Michael Didierjean, Salima Domrane, Brigitte Douzou, Sabine Druart, Xavier Dubeau, Arnaud Dubedat, Résika Dudragne, Christian Duez, Lucien Dufour, Sylvie Duhamel, Lionel Duisit, Nathalie Dumoitier, Hervé Dumond, Julien Dumortier, Emilie Duquesne, David Durand, William Durieux, Philippe Durot, Taous Duss, Bénédicte Eschalier, Eric Espiard, Pierre Eterstein, Joël Etienney, Xavier Faidix, Patrice Famery, David Faria, Renaud Faure, André Ferrer, Jean-Marie Ferrer, Jean Feuillet, Christian Fivel, Christian Flaissier, Julien Fortané, Jenny Forté, Claude Fossé, Joël Foucat, Christophe Fouillard, Vianney Fournier, Virginie Fournier, Déborah Fraizy, Paul Frappé, Isabelle Frenoy-Sansarricq, Bernard Gabbai, Yoann Gaboreau, Fabien Gaillard, Isabelle Garnier, Jean-Michel Garnier, Jean-Claude Gascoin, Yves Gault, Martine Gaultier, Bruno Gay, Benoît Gédon, Christophe Genies, Jean-Marc Géniole, Jean-Luc Gentner, Jean-Louis Ghez, Charles Giraldi, Christian Girard, Philippe Giraud, Sylvain Godart, Pierre Goidin, Aline Gomez, Céline Goncalves, Arnaud Gouget, Marie-Laure Gouget, Sylvain Gournay, Florence Grand, Jean-Claude Granier, Roland Greffe, Sabine Grutter, Catherine Gryb, François-Xavier Guedel, Mathieu Guérin, Julien Guiberteau, Jean-François Guille, Jean-Pierre Guillot, Philippe Guillou, Gilles Gustin, Anne Guyot, Jean-Marie Guyot, Christophe Hardy, David Hassid, Latifa Hayani, Anne Heller, Frédéric Henriot, Thierry Hermouet, Bénédicte Hoenner Hecht, Ferreol Honvoh Senadjro, Gaëtan Houdard, Isabelle Huas-Suarez, Corinne Huber, Patrick Imbert, Pascal Jacques, Cécile Jacquet, Jean-Pierre Jacquet, Denis Jacquiot, Philippe Jacquot, Samy Jaffre, Clémence Jean, Pauline Jeanmougin, Isabelle Jeudy, Jean-Philippe Joseph, Pierre Jouannic, Stéphanie Jousson, André Kastelik, Gérard Klifa, François-René Knockaert, Jean-Philippe Koch, Julia Krotoff, Charlotte Labrune, Véronique Lacaille-Smerilli, François Lacoin, Xavier Lainé, Philippe Lambert, Audrey Lambourg, Dominique Lamy, Myriam Lapenne-Creusot, Jean-Dominique Laporte, Lucie Lartaud, Nadège Lauchet, Dorothée Lavielle, Julien Le Breton, Raphaël Le Diagon, Marie Le Du, Delphine Le Goff, Claire Le Lann, Stéphane Le Mouël, Carole Leblan-Depelsenaire, Frédéric Leclercq, Rudolphe Lécutier, Jean-Nicolas Ledoux, Jean-Marc Lefebvre, Pascal Léger, Robert Lendais, Eric Lengagne, Catherine Sosiewicz-Lengsavath, Sylvie Lenoir, Patrick Lerouge, Benoît Leroy, Ida Leung-Y-Tai, Pascal Ligier, Charlotte Lobel, François Loez, Béatrice Lognos, Baptiste Luaces, Laurence Lucas-Couturier, Anne Lunven, Sigolène Machraoui, André Maciejewski, Loïc Magnen, Georgios Makridis, Jean-Luc Malbrunot, Jean-Marc Mancini, Hervé Mangin, Jean-Marc Maniglier, Philippe Marchant, Julie Marcus, Guillaume Marien, Anne-Laure Martin-Etzol, Christian Mas, Yannick Masset, Pierre Massin, Bruno Masson, Agnès Mattera, Charlotte Matz, François Maufoy, Collette Maury, Laure-Emmanuelle Mavraganis, Olivier Mazin, Lison Mazué, Jean-Philippe Melizan, Dominique Menard, Alain Mercier, Patricia Mercier, Patric-Alain Meyer, Philippe Michellier, Corinne Milleret, Yannick Millot, Françoise Minard, Marie-Léa Miqueu, Dany Mismacque, Catherine Mitifiot, Jean-Louis Moebs, Jean-Michel Monnier, Yves Montariol, Jérémie Montauze, Alain Morand, Gilles Morel, François Morlon, Delphine Mortas, Baptiste Motte, Stéphane Mouget, Luc Munro, Martin Naessens, Bounthanousone Nammathao, Dominique Negre, Charlie Nogrel, Lucile Nouvellet, Ismaël Nureni Banafunzi, Thierry Oger, Jean-François Ortholan, Dominique Osty, Agnès Oude Engberink, Guy-Marc Paillard, Laurent Paillard, Marie-Paule Pautout-Guillaume, Laurent Pech-Gourg, Corinne Perdrix, Véronique Perez, Ségolène Perrillat-Amédé, Christophe Peyrou, Bruno Pichat, Christophe Pigache, Alexis Pinot, Dominique Piquard, Thierry Piquet, Michel Placet, Michel Plauchier, Caroline Pluskota, Maurice Ponchant, Chantal Prat, Martine Prevost, Jean-Pierre Prigent, Thibaut Py, Christian Rafin, Jacques Rambaud, Jean-Paul Rapoud, Anne-Marie Regnier, Sylvain Renaudin, Jean-Michel Rétaux, Amélie Richard, Philippe Richetta, Jean-Michel Rigault, Reinold Rigoli, Anne Ritter-Meinicke, Sarah Robert, Stéphanie Rollin, Didier Rondepierre, Sophie Rosenberg, Mélanie Roth, Fabien Rougerie, Guillaume Royer De Vericourt, Karen Rudelle, Philippe Ruelle, Marcel Ruetsch, Dominique Saillard, Pénélope Saint-Denis, Pietro Sannelli, Philippe Saraidarian, Jean-Pascal Sastourné, Laurent Sauvage, Christian Scellier, Christian Schaal, François-Xavier Schelcher, Daniel Schirlin, Anne Schirrer, Claude Schlienger, Joëlle Schlienger, Philippe Serayet, Denis Serramoune, Marlène Siebler, Jean-Paul Simon, André Soares, Carine Soussotte-Ducasse, Philippe Stefanuto, Marc Steinberger, Marianne Szapiro, Anas Taha, Erol Taluy, Gilles Tanguy, Dominique Tardieux, Michel Tardy, Benoît Tavernier, Jean-Luc Ténédos, Lorène Thelot-Bach, Rémy Tisserand, Audrey Tordoir, François Trillot, Pascal Triouleyre, David Truong, Laurent Turi, Frédéric Vaillant, Hélène Vaillant-Roussel, Pierre Vailler, Josette Vallee, Muriel Vampouille, Jean-Louis Vangi, Fabien Vannier, Simon Varin, Florence Vaugeois, Jean-Charles Vauthier, Virginie Vauthier, Delphine Veillard, Anne-Laure Verjus, Paul-Bernard Verjus, Gilles Verney, Eloïse Vialtel, Fernand Vierling, Graziella Virgone-Rebaud, Marc Vital-Durand, Eric Vittori, Nadège Volcler, Philippe Vorilhon, Pierre Watteau, Christine Weisbecker, Nathalie Wey, Françoise Wilhelm-Nenot, Jean-Louis Wurtz, Patricia Yvon, Claire Zabawa, Jean-Marc Zamboni, Anne-Claire Zipper

## Abbreviations

ADR: adverse drug reaction
DOAC: direct oral anticoagulant
VKA: vitamin-k antagonist
ESC: European Society of Cardiology
